# Contribution to the Study of Mallein

**Published:** 1892-02

**Authors:** W. Dieckerhoff, R. Lothes

**Affiliations:** Royal Veterinary School, Berlin; Royal Veterinary School, Berlin


### CONTRIBUTION TO THE STUDY OF MALLEIN.

Prof. Dr. W. Dieckerhoff and Dr. R. Lothes, Royal Veterinary School, Berlin. Translated by Leonard Pearson, B. S., V. M. D., Veterinary Department, University of Pennsylvania.

After Preusse published the results of his first experiments with mallein, we were requested by the Government to conduct a series of experiments with this substance, which was sent to us by Preusse. These experiments are not yet completed, and for this reason we must withhold our final decision, but on account of the general interest which the use of mallein, in diagnosing obscure and concealed cases of glanders, has awakened, we think it advisable to publish the results of our investigations so far as they have gone.

We commenced our experiments, with Preusse's mallein, on guinea pigs, and found that all glanderous animals reacted after the subcutaneous injection of 0.5 c. c. of the glanderous lymph, while the temperature rose 1° C.; while in healthy animals the same treatment caused nothing but a scarcely noticeable change of the temperature; we are, therefore, able to confirm the results obtained by Dr. Pearson in his experiments on animals of this species. The following table clearly shows the effect on the temperature:

Healthy Guinea-pig.

Inoculated August 21st, 6 a. m, with 0,5 grm. of mallein.

| Time, | 6 a m | 8 a m | 10 a m | 12 a m | 2 p m | 4 p m | 6 p m | 8 p m |
|---|---|---|---|---|---|---|---|---|
| Temperature, | 38.6 | 38.7 | 38.7 | 38.6 | 38.6 | 39.0 | 38 9 | 38.7 |

Glanderous Guinea-pig.

Inoculated September 5th, 7 a m, with 0,5 grm. of mallein.

| Time, | 7 a m | 9 a m | 11 a m | 1 p m | 3 p m | 5 p m | 7 p m | 9 p m |
|---|---|---|---|---|---|---|---|---|
| Temperature, | 38.9 | 39.7 | 40,4 | 39.6 | 39.5 | 39.2 | 39.2 | 39.1 |

In the stable of the livery man *Sch.* in *R.*, containing 75 horses glanders was discovered in one horse July 6th, 1890. The remaining 74 horses were kept under obsorvation, and 17 of them died before the 23d of August, 1891. The post-mortem examinations showed but one of these horses to have suffered from glanders. Aside from this, 38 horses were, from time to time, killed by order of the police, and the autopsies showed each of them to have suffered from glanders.

When we commenced our experiments, the 23d of August, this year, 20 horses still remained in this stable, Of these, one clear case of glanders received but one injection, but all of the other horses were inoculated with mallein from two to five times. During the experiments, the results of which are given below, three horses showed symptoms strongly suspicious of glanders and were killed and autopsies made by the order of the local police, and in all cases the suspicion of glanders was confirmed. The remaining 16 horses were killed, at the end of our experiments, on the 28th, 29th, and 30th of September. Thirteen of them were found to have been healthy and three were suffering from glanders.

Mallein is an amber colored, clear and easily flowing liquid, and that used by us was diluted every day before the experiment with 9 parts water, containing from 1 to 2 per cent. carbolic acid. In the application of the lymph prepared in this way, which was nearly as clear as water, we used a Koch inoculation syringe, which was always carefully disinfected before and after use. The injections were made, in every case, under the skin of the neck, which was always thoroughly cleaned with creoline soap and a brush and rinsed off with corrosive sublimate solution. In most of the experiments we injected 5 grm. of diluted mallein into the subcutis, but this dose was sometimes increased after a number had been given. In many of the horses a small inflammatory swelling, from the size of a walnut to that of the fist, appeared within the first 24 hours after the injection, and disappeared during the following day. The post-mortems were made, according to the legally prescribed method, by the district veterinarian, Klein. These autopsies were made in our presence and the presence of a number of practicing veterinarians, and there was not, in a single case, a difference of opinion as to the discoveries.

*Horse No. 1.* Iron gray mare, 7 years old, good condition, and has been in perfect health according to the report of the owner. A careful examination was made before the injection but no symptoms of disease were discoverable. An inflammatory swelling, the size of the fist, which developed after the third inoculation did not disappear until fourteen days had elapsed. The horse was killed on the 30th of September. The autopsy revealed numerous glanderous nodules in the lungs, from the size of lentil to that of a pea, All of these nodules were surrounded by an intensely red inflammatory zone, ¼ to ½ of a cm. broad. Aside from this, there were a number of centres of inflammation in the lungs, from the size of a walnut to that of an apple. Bronchial glands were enlarged.

First inoculation August 23d, 7.45 a m, with 0,5 grm. of mallein.

| | | | | | | | | | | | |
|---|---|---|---|---|---|---|---|---|---|---|---|
| Time, | 7.45 a m | 10 | 11.15 | 1.15 p m | 3 | 4.15 | 5.30 | 7 | 8.30 | 10 | 11.30 |
| Pulse, | 45 | 47 | 45 | 43 | 43 | 42 | 46 | 45 | 48 | 47 | 49 |
| Respiration, | 19 | 20 | 18 | 21 | 24 | 26 | 24 | 22 | 26 | 24 | 23 |
| Temperature, | 38.1 | 38.4 | 38.4 | 38.3 | 38.3 | 38.7 | 38.6 | 38.9 | 38.8 | 39 | 39.6 |

Second inoculation September 1st, 8 a m. 0.5 grm. mallein.

| | | | | | | | | | | | | | | |
|---|---|---|---|---|---|---|---|---|---|---|---|---|---|---|
| Time, | 8 a m | 9.30 | 10.30 | 11.45 | 1 p m | 2 | 3.15 | 4.30 | 6 | 8 | 9.30 | 11 | 12.30 a m | 4.30 |
| Pulse, | 38 | 42 | 40 | 40 | 38 | 43 | 45 | 45 | 54 | 53 | 54 | 57 | 57 | |
| Respiration, | 15 | 13 | 16 | 15 | 15 | 16 | 12 | 14 | 17 | 27 | 29 | 28 | 27 | 27 |
| Temperature, | 37.6 | 37.6 | 37.6 | 37.5 | 38.2 | 38.8 | 39.1 | 39.6 | 40.1 | 40.3 | 39.8 | 39.6 | 39.4 | 38.7 |

Third inoculation September 6th, 7 a m, 0,5 grm mallein.

| | | | | | | | | | | | | |
|---|---|---|---|---|---|---|---|---|---|---|---|---|
| Time, | 7 a m | 8.30 | 10 | 11.30 | 1 p m | 2.30 | 4 | 5.30 | 7 | 8.30 | 10 | 11.30 |
| Pulse, | 42 | 40 | 40 | 42 | 48 | 45 | 45 | 43 | 42 | 45 | 44 | 45 |
| Respiration, | 24 | 25 | 24 | 19 | 18 | 24 | 24 | 22 | 20 | 25 | 23 | 25 |
| Temperature, | 38.5 | 38.2 | 38. | 38.3 | 38.5 | 38.5 | 38.5 | 38.4 | 38.7 | 39.2 | 39.2 | 39.2 |

Fourth inoculation September 27th, 7.45 a m, 0.5 grm. mallein.

| | | | | | | | | | |
|---|---|---|---|---|---|---|---|---|---|
| Time. | 7.45 a m | 9.30 | 11 | 1 p m | 3 | 5 | 7 | 9 | 11 |
| Pulse, | 36 | 36 | 37 | 38 | 36 | 36 | 38 | 36 | 37 |
| Respiration, | 14 | 13 | 13 | 13 | 11 | 12 | 13 | 11 | 12 |
| Temperature, | 37.7 | 38.3 | 38.3 | 38.2 | 37.9 | 37.9 | 38.5 | 38.4 | 38.7 |

Fifth inoculation September 28th, 8.30 a m, 0.75 grm. mallein.

| | | | | | | | | | | |
|---|---|---|---|---|---|---|---|---|---|---|
| Time, | 8.30 a m | 10.30 | 12.30 p m | 2 30 | 4.30 | 6.30 | 8.30 | 10.30 | 11.45 | 4.30 a m |
| Pulse, | 36 | 36 | 40 | 40 | 44 | 44 | 44 | 52 | 68 | |
| Respiration, | 10 | 16 | 16 | 16 | 16 | 16 | 16 | 14 | 18 | |
| Temperature, | 37.9 | 38.1 | 38.5 | 38.3 | 38.0 | 38.4 | 38.8 | 40.5 | 39.8 | 39.3 |

*Horse No. 2.* Sorrel gelding, 12 years old, in medium condition. Before the injection, there was a discharge of grayish-white, sero-mucous material from the left nostril. After the injection the horse lost his appetite, stood back from the manger, and became dull. After the second inoculation the animal developed a frequent short, hoarse cough.

It was killed and the autopsy made on the 30th of September. The autopsy revealed glanderous nodules from the size of a hemp-seed to that of a hazel-nut in both lungs, but, especially in the left one. Some of these nodules were superficial, and the pleura covering them was swollen and opaque. Nodules, of the size of a grain of rice, were found in the liver. There were a number of partially cicatrised nodules in the mucous membrane of the epiglottis.

First inoculation August 23d, 7.30 a m, 0.5 grm. mallein.

| | | | | | | | | | | | |
|---|---|---|---|---|---|---|---|---|---|---|---|
| Time, | 7.30 a m | 10 | 11.30 | 1.30 p m | 3 | 4.30 | 5.30 | 6.30 | 8.30 | 10 | 11.30 |
| Pulse, | 47 | 50 | 46 | 45 | 45 | 46 | 45 | 55 | 47 | 52 | 49 |
| Respiration, | 14 | 13 | 14 | 15 | 13 | 14 | 14 | 22 | 21 | 19 | 13 |
| Temperature, | 38.1 | 38.1 | 38.1 | 38.2 | 38. | 38.3 | 38. | 39.1 | 39.6 | 39.9 | 39.4 |

Second inoculation September 1st, 8.15 a m, 0.5 grm. mallein.

| | | | | | | | | | | | | | | |
|---|---|---|---|---|---|---|---|---|---|---|---|---|---|---|
| Time, | 8.15 a m | 9.30 | 10 45 | 12 | 1 p m | 2 15 | 3.30 | 5 | 6.15 | 8 | 9.30 | 11 | 12.30 | 4.45 a m. |
| Pulse, | 48 | 47 | 47 | 45 | 44 | 46 | 45 | 43 | 47 | 46 | 46 | 46 | 46 | |
| Respiration, | 14 | 14 | 13 | 12 | 13 | 12 | 11 | 12 | 14 | 18 | 18 | 18 | 17 | 16 |
| Temperature, | 37.7 | 38. | 37.5 | 37.5 | 37.8 | 37.7 | 37.9 | 37.7 | 38. | 38. | 37.9 | 38.1 | 38 8 | 37.5 |

Third inoculation, September 6th, 7 a m, 0.5 grm. mallein.

| | | | | | | | | | | | |
|---|---|---|---|---|---|---|---|---|---|---|---|
| Time, | 7 a m | 8.30 | 10 | 11.30 | 1 p m | 2.30 | 4 | 5.30 | 7 | 8.30 | 10 |
| Pulse, | 48 | 47 | 48 | 48 | 46 | 48 | 48 | 48 | 49 | 51 | 50 |
| Respiration, | 16 | 16 | 17 | 16 | 24 | 18 | 17 | 16 | 16 | 16 | 16 |
| Temperature, | 38.3 | 38.2 | 37.9 | 38.1 | 38.2 | 38.3 | 38.1 | 38.3 | 38.2 | 38.2 | 38.1 |

Fourth inoculation, September 28th, 8.15 a m, 0.75 grm. mallein.

| | | | | | | | | | | |
|---|---|---|---|---|---|---|---|---|---|---|
| Time, | 8.30 a m | 10.30 | 12.30 p m | 2.30 | 4.30 | 6.30 | 8.30 | 10.30 | 12 | 4.30 a m. |
| Pulse, | 37 | 36 | 38 | 40 | 48 | 52 | 48 | 44 | 44 | |
| Respiration, | 15 | 16 | 16 | 16 | 16 | 19 | 20 | 20 | 18 | 16 |
| Temperature, | 37.1 | 37.7 | 37.5 | 37.9 | 38.0 | 38.6 | 38.3 | 38.2 | 38. | 37.8 |

*Horse No. 3.* Dark-brown mare, 13 years old, coat staring, but condition of animal good. Killed on the 30th of September and an autopsy made. Both lungs contained numerous glanderous nodules of the size of a lentil seed. Some of them were surrounded by small, fresh inflammatory zones. The bronchial glands were enlarged and contained large numbers of small glanderous nodules. There were a number of star-shaped scars from the size of a pea to that of a cent, on the mucous membrane of the nasal septum and of the turbinated bones.

First inoculation, August 23d, 7.45 a m, 0.5 grm. mallein.

| | | | | | | | | | | | |
|---|---|---|---|---|---|---|---|---|---|---|---|
| Time, | 7.45 a m | 9.30 | 11.15 | 1.15 p m | 3 | 4.15 | 5.30 | 6.45 | 8.15 | 10 | 11.15 |
| Pulse, | 43 | 42 | 43 | 44 | 54 | 48 | 46 | 47 | 48 | 48 | 49 |
| Respiration, | 12 | 19 | 20 | 19 | 22 | 22 | 21 | 22 | 23 | 21 | 21 |
| Temperature, | 37.7 | 38.2 | 38. | 38.3 | 38 2 | 38.3 | 38. | 39.2 | 37.9 | 37.8 | 37.8 |

Second inoculation, September 27th, 8 a m, 0.5 grm. mallein.

| | | | | | | | | | |
|---|---|---|---|---|---|---|---|---|---|
| Time | 8 a m | 9.30 | 11 | 1 p m | 3 | 5 | 7 | 9 | 11 |
| Pulse, | 37 | 36 | 38 | 39 | 39 | 38 | 44 | 45 | 56 |
| Respiration, | 14 | 14 | 13 | 13 | 13 | 14 | 15 | 12 | 14 |
| Temperature, | 38 2 | 38.4 | 38. | 38.2 | 38.1 | 38.3 | 38.2 | 38.4 | 38.1 |

Third inoculation, September 28th, 8.30 a m, 1.0 grm. mallein.

| | | | | | | | | | | |
|---|---|---|---|---|---|---|---|---|---|---|
| Time, | 8.30 a m | 10.30 | 12.30 p m | 2.30 | 4.30 | 6.30 | 8.30 | 10.30 | 12 | 4.30 a m. |
| Pulse, | 43 | 44 | 52 | 52 | 60 | 64 | 60 | 60 | 60 | |
| Respiration, | 12 | 12 | 16 | 16 | 16 | 16 | 16 | 19 | 22 | 19 |
| Temperature, | 38.6 | 39. | 39.2 | 38.9 | 39.5 | 39.9 | 39.9 | 39.7 | 39.7 | 39.4 |

*Horse No. 4.* Dark-brown gelding, 10 years old, apparently healthy before the first injection. On the day of the second injection a flow of serous material commenced from the right nostril, while a mass of sero-mucous material was discharged from the left nostril. The inter-maxillary lymph glands on the left hand side were as large as a walnut, firm, painless, and attached to the skin. Horse was killed on the 7th of September and the autopsy made the same day. It was found that there were glanderous foci, from the size of a walnut to that of the fist, in both lungs. Aside from this, large numbers of glanderous scars were found in the mucous membrane of the larynx and a catarrhal inflammation of the nasal mucous membrane.

First inoculation, August 23d, 7.30 a m, 0.5 grm. mallein.

| | | | | | | | | | | | |
|---|---|---|---|---|---|---|---|---|---|---|---|
| Time, | 7.30 a m | 10 | 11.30 | 1.30 p m | 3 | 4.15 | 5.30 | 6.30 | 8.30 | 10 | 11.30 |
| Pulse, | 37 | 46 | 45 | 38 | 39 | 37 | 38 | 39 | 38 | 38 | 38 |
| Respiration, | 16 | 16 | 17 | 17 | 17 | 18 | 18 | 19 | 22 | 22 | 23 |
| Temperature, | 37.7 | 37.9 | 38.1 | 37.9 | 38. | 38.2 | 37.6 | 38. | **39.9** | 38.4 | 38.1 |

Second inoculation, September 6th, 7 a m, 0.5 grm. mallein.

| | | | | | | | | | | | |
|---|---|---|---|---|---|---|---|---|---|---|---|
| Time, | 7 a m | 8.30 | 10 | 11.30 | 1 p m | 2.30 | 4 | 5.30 | 7 | 8.30 | 10 |
| Pulse, | 41 | 45 | 44 | 42 | 43 | 42 | 44 | 43 | 43 | 43 | 42 |
| Respiration, | 19 | 19 | 18 | 17 | 17 | 18 | 20 | 19 | 17 | 17 | 17 |
| Temperature, | 37.9 | 37.9 | 38. | 38.4 | **39.4** | 38.4 | 38.2 | 37.8 | 37.7 | 37.8 | 37.9 |

Third inoculation, September 27th, 8 a m 0.5 grm. mallein.

| | | | | | | | | | |
|---|---|---|---|---|---|---|---|---|---|
| Time, | 8 a m | 9.30 | 11 | 1 p m | 3 | 5 | 7 | 9 | 11 |
| Pulse, | 48 | 40 | 44 | 43 | 42 | 41 | 44 | 41 | 45 |
| Respiration, | 14 | 12 | 18 | 18 | 12 | 12 | 11 | 13 | 13 |
| Temperature, | 38.2 | 38.1 | 37.9 | 37.9 | 37.7 | 37.7 | 38.1 | 38.2 | 37.9 |

*Horse No. 5.* Brown-gelding, 7 years old, in medium condition. Bilateral grayish-white, tough mucous nasal discharge before the first injection. After the second injection, an increased vesicular murmur could be heard in the upper portion of both lungs. The horse was killed and examined on the 7th of September, and it was found that there were numerous glanderous nodules, from the size of a grain of rice to that of a pea, in both lungs. Some of these were quite fresh, but some were softened at the centre. The pleura presented numerous thickened areas of the size of a dollar. There were some glanderous nodules, from the size of a millet-seed to that of a pea, in the liver. A glanderous patch as large as a walnut was found in the spleen.

First inoculation, August 23d, 8 a m, 0.5 grm mallein.

| | | | | | | | | | | | |
|---|---|---|---|---|---|---|---|---|---|---|---|
| Time, | 8 a m | 10 | 11.15 | 1.15 p m | 3 | 4.15 | 5.15 | 6.30 | 8.30 | 10 | 11 |
| Pulse, | 39 | 40 | 40 | 42 | 46 | 44 | 47 | 44 | 48 | 63 | 62 |
| Respiration, | 19 | 22 | 18 | 28 | 29 | 24 | 26 | 24 | 26 | 29 | 27 |
| Temperature, | 38.1 | 37.8 | 38.1 | 38.5 | 38.5 | 38.9 | 39.0 | 38.5 | **39.0** | **39.6** | **39.5** |

Second inoculation, September 1st, 8 a m, 0.5 grm. mallein.

| | | | | | | | | | | | | | |
|---|---|---|---|---|---|---|---|---|---|---|---|---|---|
| Time, | 8 a m | 9.45 | 10.45 | 12 | 1.15 p m | 2.15 | 3.45 | 5.15 | 6.45 | 8.30 | 9.45 | 11.15 | 12.45 a m | 4.45 |
| Pulse, | 40 | 40 | 39 | 39 | 42 | 42 | 43 | 44 | 52 | 56 | 54 | 61 | | |
| Respiration, | 29 | 29 | 25 | 28 | 28 | 27 | 28 | 28 | 23 | 24 | 26 | 25 | 28 | 28 |
| Temperature, | 38.2 | 38.3 | 38.3 | 38.2 | 38.6 | 38.6 | 38.5 | 38.6 | 38.5 | 39.1 | **39.6** | **39.4** | **40.5** | **40.5** |

Third inoculation, September 6th, 7.30 a m, 0.4 grm. mallein.

| | | | | | | | | | | | |
|---|---|---|---|---|---|---|---|---|---|---|---|
| Time, | 7.30 a m | 9 | 10 | 11.30 | 1 p m | 2.30 | 4 | 5.30 | 7 | 8.30 | 10 |
| Pulse, | 44 | 48 | 42 | 44 | 46 | 46 | 47 | 46 | 52 | 49 | 49 |
| Respiration | 2 | 20 | 22 | 24 | 30 | 32 | 29 | 31 | 31 | 29 | 31 |
| Temperatu.e, | 39.4 | 39.2 | 39.8 | 39.7 | 39.6 | 39.7 | 39.5 | 39.4 | 39.5 | 39.4 | 39.1 |

*Horse No 6.* Iron gray mare, 7 years old, in poor condition, mate of horse No. 1. Mucous membranes of the head pale red, collection of grayish-yellow tough mucous in inner angle of the eye. The left inter-maxillary gland was as large as a pigeon's egg, firm and not tender to pressure, and not attached to the skin. On the third day after the first injection, this gland became more swollen until, on the fifth day, it reached the size of a hen's egg and was sensitive to pressure. On the sixth day, after the first injection, the right inter-maxillary lymphatic gland became as large as a walnut At the same time a grayish-yellow nodule of the size of a pea was found in the mucous membrane of the wing of the left nostril, and a slightly clouded serous fluid escaped from both nasal orifices. The second injection was made on the seventh day after the first. The nodules in the nasal mucous membrane had already commenced to break down, and the flow from the left nostril was of a grayish-white color and of the consistency of tough mucous. A few hours after the second injection a second nodule, about as large as a grain of rice, was discovered immediately above the first one.

The horse was killed and the autopsy made on the 2d of September. Six nodules of the size of a bean were found in the spleen. Innumerable glanderous nodules, from the size of a millet-seed to that of a lentil, were found scattered through the lungs. The blunt border of the left lung presented a spot of glanderous pneumonia as large as a man's fist. The bronchial glands were swollen. The mucous membrane of the middle portion of the trachea, the larynx, the nasal septum, and the left superior turbinated bone, were covered with glanderous ulcers, from the size of a bean to that of a walnut, and numerous fresh milliary nodules were found around their borders. The inter-maxillary glands of the right hand side were as large as a pigeon's egg. The corresponding glands of the left hand side had attained the size of a plum, and the slightly swollen inguinal glands contained numerous milliary nodules of varying ages.

First inoculation, August 23d, 7.45 a m, 0.5 grm. mallein.

| | | | | | | | | | | | |
|---|---|---|---|---|---|---|---|---|---|---|---|
| Time, | 7.45 a m | 10 | 11.15 | 1.15 p m | 3 | 4.15 | 5.15 | 6.30 | 8.15 | 10 | 11 |
| Pulse, | 45 | 52 | 52 | 44 | 56 | 55 | 56 | 60 | 57 | 60 | |
| Respiration, | 11 | 13 | 16 | 16 | 17 | 13 | 13 | 21 | 20 | 18 | 19 |
| Temperature, | 38.2 | 38.3 | 38.2 | 38.7 | 39.4 | 38.7 | 39.2 | 39.1 | 39.9 | 39.6 | 39.7 |

Second inoculation, September 1st, 8.15 a m, 0.5 grm. mallein.

| | | | | | | | | | | | | | | |
|---|---|---|---|---|---|---|---|---|---|---|---|---|---|---|
| Time | 8.15 a m | 9.30 | 10.45 | 12 | 1.15 p m | 2.30 | 4.30 | 5.30 | 6.30 | 8.45 | 9.45 | 11.15 | 12 | 4.45 |
| Pulse, | 53 | 50 | 56 | 54 | 53 | 57 | 55 | 55 | 53 | 53 | 52 | 54 | | |
| Respiration, | 14 | 15 | 17 | 17 | 16 | 16 | 11 | 10 | 10 | 11 | 12 | 12 | 13 | 13 |
| Temperature, | 37.5 | 38.8 | 38.8 | 38.9 | 39.0 | 39.0 | 39.0 | 38.6 | 38.5 | 38.9 | 38.7 | 38.8 | 39.4 | 39.4 |

*Horse No. 7.* Sorrel mare, 9 years old, in poor condition, conjunctiva dirty red, collection of yellow tough mucous in the inner angles of the eye. There was a discharge of a small quantity of grayish-white sticky mucous from the nostril. Inter-maxillary lymph glands of the left hand side were high up and somewhat enlarged. There was a farcinous swelling of the size of a saucer on the left side of the thorax, and one the size of a hen's egg on the right. The latter was broken through in the middle and discharged a cloudy grayish-yellow fluid, which dried and formed dirty yellowish-brown crusts around the opening. The skin on the left hind leg was thickened and contained numerous "farcy-buds," from the size of a hazel-nut to that of an apple, some of which had broken and discharged a dirty grayish-yellow pus. The lymph vessels on this leg were corded and above the hock, as well as in the region of the inguinal ring, presented irregular hard swellings along their course. The inguinal glands on the left hand side were somewhat enlarged and firm. About ten hours after the injection, the "farcy-buds" in the skin, and especially the one on the right chest wall, became somewhat smaller. The horse was killed on the 24th of August and the autopsy showed glanders of the skin in the parts already mentioned. Aside from this, some twenty glanderous nodules, from the size of a pea to that of a hazel-nut, were found in the spleen, and some of them were surrounded by a firm connective tissue capsule. The lungs contained numerous nodules, from the size of a lentil to that of a bean, some of them were incapsulated, and in some the centres were broken down into a soft mass. The bronchial glands were enlarged. The mucous membrane of the upper air passages presented no specific alterations.

First inoculation, August 23d, 8 a m, 0.5 grm. mallein.

| | | | | | | | | | | |
|---|---|---|---|---|---|---|---|---|---|---|
| Time, | 8 a m | 10.15 | 11.15 | 1.15 p m | 3 | 4.15 | 5.15 | 6.30 | 8.15 | 10 |
| Pulse, | 52 | 55 | 56 | 59 | 60 | 53 | 53 | 50 | 55 | 54 |
| Respiration, | 14 | 14 | 14 | 15 | 15 | 12 | 13 | 14 | 12 | 14 |
| Temperature, | 38.6 | 39.1 | 29.0 | 39.4 | 39.5 | 39.6 | 39 5 | 39.5 | 39.9 | 39.5 |

*Horse No. 8.* Dun gelding, 10 years old. Aside from the long, rough hair and poor condition, the animal presented no symptoms of disease. Immediately after the third injection, on the 28th of September, a short oppressive cough commenced, which remained until the killing of the animal two days later. At the post-mortem we found nothing but a small grayish-red nodule, of the size of a lentil, surrounded by a dark-red zone, in the anterior lobe of the left lung. As this lesion was not sufficiently characteristic to justify a diagnosis of glanders, a guinea-pig was inoculated with the nodule on the day of the post-mortem, but did not become diseased, and a post-mortem was made upon it the 8th of December; it was found to be healthy.

First inoculation, September 1st, 8 a m, 0.5 grm. mallein.

| Time, | 8 a m | 10 | 11.15 | 1.30 p m | 3 | 4.15 | 5.30 | 6.45 | 8.30 | 10 | 11 |
|---|---|---|---|---|---|---|---|---|---|---|---|
| Pulse, | 42 | 44 | 53 | 49 | 56 | 47 | 50 | 47 | 46 | 45 | 43 |
| Respiration, | 19 | 21 | 20 | 19 | 18 | 18 | 19 | 21 | 25 | 24 | 26 |
| Temperature, | 37.7 | 38.3 | 38.6 | 38.3 | 38.2 | 37.7 | 38.5 | 37.9 | 37.9 | 37.8 | 37.9 |

Second inoculation, September 27th, 8 a m, 0.5 grm. mallein.

| Time, | 8 a m | 9.30 | 11 | 1 p m | 3 | 5 | 7 | 9 | 11 |
|---|---|---|---|---|---|---|---|---|---|
| Pulse, | 40 | 40 | 42 | 44 | 38 | 36 | 45 | 41 | 40 |
| Respiration, | 16 | 13 | 15 | 14 | 13 | 14 | 14 | 13 | 14 |
| Temperature, | 38.1 | 38.1 | 38.0 | 38.0 | 37.9 | 37.9 | 38.1 | 38.1 | 37.8 |

Third inoculation, September 28th, 8.30 a m, 0.75 grm. mallein.

| Time, | 8.30 a m | 10.30 | 12.30 p m | 2.30 | 4.30 | 6.30 | 8.30 | 10.30 | 11.45 | 4.30 a m. |
|---|---|---|---|---|---|---|---|---|---|---|
| Pulse, | 40 | 40 | 40 | 52 | 64 | 75 | 60 | 68 | 68 | |
| Respiration, | 10 | 12 | 16 | 16 | 20 | 16 | 16 | 20 | 19 | 12 |
| Temperature, | 37.5 | 37.8 | 38.0 | 38.5 | 39.7 | 40.5 | 40.5 | 40.5 | 40.0 | 39.0 |

*Horse No. 9.* Iron-gray gelding, 9 years old, in medium condition, no symptoms of disease were discoverable. The animal was killed and an autopsy made on the 30th of September, and aside from a thickening of the left pulmonary pleura, of the size of a dollar, no alterations of disease could be found.

First inoculation, August 23d, 8.30 a m, 0.5 grm. mallein.

| Time, | 8 a m | 10 | 11.15 | 1.30 p m | 3 | 4.15 | 5.15 | 6.30 | 8.15 | 10 |
|---|---|---|---|---|---|---|---|---|---|---|
| Pulse, | 38 | 37 | 40 | 40 | 40 | 37 | 39 | 41 | 36 | 36 |
| Respiration, | 15 | 16 | 17 | 16 | 16 | 18 | 18 | 19 | 15 | 14 |
| Temperature, | 37.5 | 37.9 | 37.9 | 37.9 | 38.5 | 38.0 | 38.0 | 38.4 | 37.8 | 37.6 |

Second inoculation, September 6th, 7.30 a m, 0.5 grm. mallein.

| Time, | 7.30 a m | 9 | 10 | 11.30 | 1 p m | 2.30 | 4 | 5.30 | 7 | 8.30 | 10 |
|---|---|---|---|---|---|---|---|---|---|---|---|
| Pulse, | 38 | 38 | 38 | 38 | 36 | 36 | 39 | 36 | 39 | 37 | 36 |
| Respiration, | 19 | 15 | 19 | 21 | 18 | 14 | 14 | 14 | 15 | 14 | 17 |
| Temperature, | 37.6 | 38.1 | 37.8 | 37.9 | 37.9 | 37.9 | 37.9 | 37.9 | 37.8 | 38.3 | 37.8 |

Third inoculation, September 27th, 7.30 a m, 0.5 grm. mallein.

| Time, | 7.30 a m | 9.30 | 11 | 1 p m | 3 | 5 | 7 | 9 | 11 |
|---|---|---|---|---|---|---|---|---|---|
| Pulse, | 40 | 48 | 48 | 48 | 40 | 37 | 36 | 38 | 36 |
| Respiration, | 13 | 15 | 15 | 12 | 13 | 11 | 10 | 10 | 10 |
| Temperature, | 38.3 | 38.0 | 37.9 | 37.8 | 37.6 | 37.8 | 38.2 | 38.2 | 37.8 |

Fourth inoculation, September 28th, 8.30 a m, 0.75 grm. mallein.

| Time, | 8.30 a m | 10.30 | 12.30 p m | 2.30 | 4.30 | 8.30 | 10.30 | 11.45 | 4.30 a m. |
|---|---|---|---|---|---|---|---|---|---|
| Pulse, | 38 | 38 | 37 | 36 | 36 | 36 | 37 | 37 | |
| Respiration, | 11 | 11 | 12 | 11 | 10 | 10 | 10 | 10 | 10 |
| Temperature, | 38.0 | 37.9 | 37.9 | 37.8 | 37.9 | 38.0 | 38.0 | 38.0 | 37.8 |

*Horse No. 10.* Sorrel mare, 11 years old, in good condition, no symptoms of disease were found either before or after the injection. On the 28th of September the horse was killed and the autopsy revealed no glanderous lesions.

First inoculation, August 23d, 8.45 a m, 0.5 grm. mallein.

| | | | | | | | | | | |
|---|---|---|---|---|---|---|---|---|---|---|
| Time, | 8.45 a m | 10 | 11.15 | 1.15 p m | 3 | 4.15 | 5.30 | 6.45 | 8.15 | 10 |
| Pulse, | 44 | 45 | 42 | 41 | 46 | 42 | 46 | 45 | 44 | 44 |
| Respiration, | 14 | 16 | 14 | 15 | 17 | 16 | 16 | 16 | 15 | 17 |
| Temperature, | 38.6 | 38.1 | 38.3 | 38.2 | 38.7 | 38.0 | 38.2 | 38.2 | 38.1 | 38.0 |

Second inoculation, September 6th, 7 a m, 0.5 grm. mallein.

| | | | | | | | | | | |
|---|---|---|---|---|---|---|---|---|---|---|
| Time. | 7 a m | 9 | 10.30 | 12.30 p m | 1.30 | 2.30 | 4 | 5.30 | 7 | 10 |
| Pulse, | 48 | 45 | 44 | 44 | 43 | 45 | 46 | 47 | 45 | 46 |
| Respiration, | 13 | 12 | 13 | 14 | 15 | 15 | 15 | 16 | 15 | 15 |
| Temperature, | 38.2 | 38.1 | 38.0 | 38.1 | 38.2 | 38.1 | 38.1 | 38.2 | 38.2 | 37.9 |

Third inoculation, September 27th, 8.15 a m, 0.5 grm. mallein.

| | | | | | | | | | |
|---|---|---|---|---|---|---|---|---|---|
| Time, | 8.30 a m | 9.30 | 11 | 1 p m | 3 | 5 | 7 | 9 | 11 |
| Pulse, | 42 | 43 | 45 | 48 | 47 | 44 | 44 | 44 | 45 |
| Respiration, | 13 | 14 | 15 | 14 | 14 | 12 | 12 | 15 | 14 |
| Temperature, | 37.7 | 37.9 | 37.6 | 38.0 | 37.7 | 37 7 | 38.1 | 37.8 | 37 7 |

*Horse No. 11.* Black gelding, 12 years old, in good condition. Examinations before and after injections revealed nothing abnormal. Horse was killed on the 29th of September, and a slight thickening of the pleura was the only abnormality to be discovered.

First inoculation, August 23d, 8.30 a m, 0.5 grm. mallein.

| | | | | | | | | | | |
|---|---|---|---|---|---|---|---|---|---|---|
| Time, | 8.30 a m | 10 | 11.15 | 1.20 p m | 3 | 4.15 | 5.15 | 6.30 | 8.15 | 10.15 |
| Pulse, | 46 | 47 | 47 | 48 | 49 | 48 | 46 | 46 | 47 | 46 |
| Respiration, | 14 | 12 | 12 | 14 | 15 | 15 | 17 | 17 | 18 | 12 |
| Temperature, | 37.7 | 38.1 | 38.0 | 38.4 | 38.6 | 38.1 | 38.1 | 38.1 | 38 2 | 38 1 |

Second inoculation, September 6th, 7 a m, 0.5 grm. mallein.

| | | | | | | | | | | | |
|---|---|---|---|---|---|---|---|---|---|---|---|
| Time, | 7 a m | 8.30 | 10 | 11 30 | 1 p m | 2 | 4 | 5 30 | 7 | 8.30 | 10 |
| Pulse. | 48 | 48 | 48 | 46 | 49 | 50 | 49 | 45 | 49 | 50 | 52 |
| Respiration, | 10 | 10 | 12 | 12 | 12 | 14 | 14 | 14 | 13 | 11 | 13 |
| Tymperature, | 38.1 | 37.8 | 37.8 | 38.1 | 38.2 | 38.0 | 38.2 | 38.0 | 37.9 | 38.0 | 38.1 |

Third inoculation, September 27th, 8.30 a m, 0.5 grm. mallein.

| | | | | | | | | | |
|---|---|---|---|---|---|---|---|---|---|
| Time, | 8.30 a m | 9 | 11 | 1 p m | 3 | 5 | 7 | 9 | 11 |
| Pulse, | 45 | 46 | 46 | 46 | 44 | 44 | 41 | 38 | 39 |
| Respiration, | 13 | 14 | 13 | 13 | 13 | 10 | 10 | 12 | 11 |
| Temperature, | 38.9 | 37.9 | 37.9 | 38.0 | 38.3 | 37.7 | 37.9 | 38.1 | 38.1 |

*Horse No. 12.* Flea bitten gray mare, 12 years old, in medium condition. Horse was killed on the 28th of September, and neither the examinations before death nor the post-mortem revealed disease.

First inoculation, August 23d, 8.15 a m, 0.5 grm. mallein.

| | | | | | | | | | | |
|---|---|---|---|---|---|---|---|---|---|---|
| Time, | 8.15 a m | 10 | 11.15 | 1.15 p m | 3 | 4.15 | 5.15 | 6.30 | 8.30 | 10.30 |
| Pulse, | 46 | 47 | 47 | 47 | 48 | 48 | 45 | 46 | 49 | 46 |
| Respiration, | 12 | 12 | 12 | 14 | 16 | 15 | 17 | 18 | 14 | 11 |
| Temperature, | 37.7 | 38.0 | 38.0 | 38.5 | 38.1 | 38.2 | 37.5 | 39.0 | 38.2 | 38.1 |

Second inoculation, September 6th, 7 a m, 5.5 grm. mallein.

| | | | | | | | | | | | |
|---|---|---|---|---|---|---|---|---|---|---|---|
| Time, | 7 a m | 9 | 10 | 11.30 | 1 p m | 2.30 | 4 | 5.30 | 7 | 8.30 | 10 |
| Pulse, | 42 | 43 | 44 | 44 | 46 | 44 | 45 | 42 | 46 | 44 | 44 |
| Respiration, | 16 | 16 | 16 | 17 | 19 | 15 | 16 | 17 | 17 | 17 | 19 |
| Temperature, | 38.1 | 37.9 | 37.7 | 37.9 | 38.3 | 38.2 | 38.3 | 38.1 | 37.9 | 38.0 | 38.1 |

Third inoculation, September 27th, 8.15 a m, 0.5 grm. mallein.

| | | | | | | | | | |
|---|---|---|---|---|---|---|---|---|---|
| Time, | 8.15 a m | 9.30 | 11 | 1 p m | 3 | 5 | 7 | 9 | 11 |
| Pulse, | 44 | 44 | 45 | 46 | 44 | 44 | 37 | 37 | 44 |
| Respiration, | 14 | 15 | 14 | 13 | 12 | 12 | 14 | 14 | 13 |
| Temperature, | 37.9 | 38.0 | 38.1 | 38.0 | 38.1 | 37.9 | 38.1 | 37.9 | 37.9 |

*Horse No. 13.* Dark-brown mare, 9 years old, in poor condition. Repeated examinations before the injections revealed nothing abnormal. The animal was killed and the autopsy made on the 28th of September, and the only abnormality discovered was an induration of the right pulmonary pleura, which was covered by numerous connective tissue filaments. The other organs were healthy.

First inoculation, August 23d, 8 a m, 0.5 grm. mallein.

| | | | | | | | | | | |
|---|---|---|---|---|---|---|---|---|---|---|
| Time, | 7.45 a m | 10 | 11.15 | 1.30 p m | 3 | 4.15 | 5.15 | 6.30 | 8.15 | 10 |
| Pulse, | 43 | 43 | 43 | 45 | 45 | 45 | 49 | 50 | 46 | 46 |
| Respiration, | 16 | 16 | 15 | 18 | 19 | 17 | 17 | 18 | 18 | 22 |
| Temperature, | 38.3 | 38.1 | 38.6 | 38.6 | 39.3 | 38.1 | 39.0 | 39.0 | 39.3 | 39.0 |

Second inoculation, September 6th, 6.30 a m, 0.5 grm. mallein.

| | | | | | | | | | | |
|---|---|---|---|---|---|---|---|---|---|---|
| Time, | 6.30 a m | 8.30 | 9.30 | 11.30 | 1 p m | 2.30 p m | 4 | 5.30 | 8.30 | 10.30 |
| Pulse, | 42 | 42 | 42 | 42 | 48 | 47 | 48 | 48 | 48 | 43 |
| Respiration, | 18 | 18 | 18 | 19 | 21 | 17 | 17 | 18 | 17 | 19 |
| Temperature, | 37.8 | 38.0 | 37.9 | 38.1 | 38.4 | 38.3 | 37.6 | 37.4 | 37.7 | 38.0 |

Third inoculation, September 27th, 8.15 a m, 0.5 grm. mallein.

| | | | | | | | | | |
|---|---|---|---|---|---|---|---|---|---|
| Time, | 8.15 a m | 9.30 | 11 | 1 p m | 3 | 5 | 7 | 9 | 11 |
| Pulse, | 42 | 41 | 44 | 45 | 45 | 39 | 40 | 37 | 37 |
| Respiration, | 13 | 12 | 14 | 13 | 15 | 13 | 16 | 14 | 15 |
| Temperature, | 38.1 | 37.9 | 37.8 | 37.9 | 37.9 | 37.7 | 38.0 | 38.1 | 37.9 |

*Horse No. 14.* Chestnut gelding, 11 years old, in medium condition. Before the first injection the horse had a phlegmonous swelling in the skin of the left front leg, which gradually disappeared on the following days. Horse was killed on the 28th of September and was found to be healthy.

First inoculation, August 23d, 8.15 a m, 0.5 grm. mallein.

| | | | | | | | | | | |
|---|---|---|---|---|---|---|---|---|---|---|
| Time, | 8 a m | 10 | 11.15 | 1.15 p m | 3 | 4.15 | 5.30 | 6.30 | 8.15 | 10 |
| Pulse, | 37 | 37 | 38 | 39 | 37 | 37 | 36 | 40 | 40 | 38 |
| Respiration, | 16 | 16 | 18 | 17 | 19 | 17 | 16 | 16 | 16 | 13 |
| Temperature, | 38.1 | 38.3 | 38.2 | 38.1 | 37.7 | 38.1 | 38.0 | 37.9 | 37.8 | 38.1 |

Second inoculation, September 27th, 8 a m, grm. mallein.

| | | | | | | | | | |
|---|---|---|---|---|---|---|---|---|---|
| Time, | 8 a m | 9.30 | 11 | 1 p m | 3 | 5 | 7 | 9 | 11 |
| Pulse, | 36 | 36 | 36 | 37 | 38 | 35 | 36 | 37 | 37 |
| Respiration, | 14 | 15 | 14 | 14 | 14 | 13 | 13 | 14 | 15 |
| Temperature, | 38.2 | 38.2 | 38.1 | 38.2 | 38.1 | 38.1 | 38.1 | 38.1 | 38.1 |

[Berliner Thierärztliche Wochenschrift, 1890, No's. 49 and 50.]

(*To be continued.*)

